# The long non-coding RNA *FOXD2-AS1* promotes bladder cancer progression and recurrence through a positive feedback loop with Akt and E2F1

**DOI:** 10.1038/s41419-018-0275-9

**Published:** 2018-02-14

**Authors:** Feng Su, Wang He, Changhao Chen, Mo Liu, Hongwei Liu, Feiyuan Xue, Junming Bi, Dawei Xu, Yue Zhao, Jian Huang, Tianxin Lin, Chun Jiang

**Affiliations:** 1 Department of Urology, Sun Yat-sen Memorial Hospital, Sun Yat-sen University, Guangzhou, Guangdong Province 510120 China; 2 Guangdong Provincial Key Laboratory of Malignant Tumor Epigenetics and Gene Regulation, Sun Yat-sen Memorial Hospital, Sun Yat-sen University, Guangzhou, Guangdong Province 510120 China; 3 Department of stomatology, Sun Yat-sen Memorial Hospital, Sun Yat-sen University, Guangzhou, Guangdong Province 510120 China; 4 Department of Osteology, Sun Yat-sen Memorial Hospital, Sun Yat-sen University, Guangzhou, Guangdong Province 510120 China; 5 Department of Gastroenterology, The First Affiliated Hospital of Sun Yat-Sen University, Guangzhou, China

## Abstract

Long non-coding RNAs (lncRNAs) have been identified as significant regulators in cancer progression. Positive feedback loops between lncRNAs and transcription factors have attracted increasing attention. Akt pathway plays a crucial role in bladder cancer growth and recurrence. In the present study, we demonstrate a novel regulatory pattern involving *FOXD2-AS1*, Akt, and E2F1. *FOXD2-AS1* is highly expressed in bladder cancer and is associated with tumor stage, recurrence, and poor prognosis. Further experiments showed that *FOXD2-AS1* promotes bladder cancer cell proliferation, migration, and invasion in vitro and in vivo. Microarray analysis demonstrated that *FOXD2-AS1* negatively regulates the expression of Tribbles pseudokinase 3 (TRIB3), a negative regulator of Akt. Mechanistically, *FOXD2-AS1* forms an RNA-DNA complex with the promoter of TRIB3, the transcriptional activity of which is subsequently repressed, and leads to the activation of Akt, which further increases the expression of E2F1, a vital transcription factor involved in the G/S transition. Interestingly, E2F1 could bind to the *FOXD2-AS1* promoter region and subsequently enhance its transcriptional activity, indicating that *FOXD2-AS1*/Akt/E2F1 forms a feedback loop. In summary, this regulatory pattern of positive feedback may be a novel target for the treatment of bladder cancer and *FOXD2-AS1* has the potential to be a new recurrence predictor.

## Introduction

Bladder cancer is one of the most common malignancies among the urothelial carcinomas worldwide. Approximately 70% of patients are diagnosed with non-muscle-invasive bladder cancer (NMIBC), which presents with a high recurrence rate^1^. According to the “seeding/implantation” theory, intraluminal seeding or intraepithelial spread results in the implantation of tumor cells in the bladder^2^. After colonization of the tumor cells, growth factors (GFs) become concentrated in the bladder mucosa, which is a major cause of the growth of bladder cancer cells^3^. Reactivation of proliferation is regarded as a significant precursor for the recurrence of bladder cancer^4, 5^. However, the underlying mechanisms that cause the proliferation of bladder cancer are still largely unknown. Therefore, to achieve a better solution to prevent bladder cancer relapse, an in-depth study of the mechanism of bladder cancer proliferation is highly necessary.

Akt, a serine/threonine kinase, plays a central role in diverse cell signaling stimuli and participates in a variety of tumor steps, including tumor growth, apoptosis, metastasis, and chemosensitivity^6^. Among these steps, tumor growth is the major progression step that is modulated by Akt. It is well known that phosphorylation of Ser473 and Thr308 is a hallmark of Akt activation^7^. Once activated, Akt can act on various downstream regulators, such as GSK3β, FOXO, and NF-κB^8^. Although positive regulation of Akt has been abundantly reported, the mechanism of negative regulation of Akt remains obscure. TRIB3 is a pseudokinase that lacks kinase activity. Thus, TRIB3 can bind to and subsequently repress phosphorylation at Thr308 of Akt. Altogether, TIRB3 serves as a significant negative modulatory factor of Akt^7^. Previous reports have revealed that the complexes formed by ATF4 and CHOP promote the expression of the TRIB3 gene by binding to a 33-bp repeating sequence in the TRIB3 promoter region^9^. However, whether there are additional vital regulators of TRIB3 remains a mystery.

LncRNAs do not code for proteins and have lengths that are greater than 200 nt and have historically been considered “transcriptional noise”. However, recent studies have shown that lncRNAs participate in multiple processes involving gene expression regulation. LncRNAs can serve either as oncogenes or tumor suppressors in cancer. Numerous studies have demonstrated that MALAT1 positively regulates proliferation, apoptosis, and metastasis of multiple cancers. Meanwhile, MEG3 is a well-known tumor suppressor that stimulates p53-mediated transactivation. A previous study found that *FOXD2-AS1* modulates tumor progression in non-small-cell lung cancer. However, whether *FOXD2-AS1* plays a role in bladder cancer is still unknown.

Despite the discovery of thousands of lncRNAs, the underlying mechanisms of their regulation are still largely unknown. Some transcription factors have been identified to regulate the expression of lncRNAs^10–12^. E2F1 belongs to the E2F family, which contains eight members that modulate diverse cell functions, such as the cell cycle, apoptosis, and the DNA damage response^13^. Ordinarily, E2F1 binds to Rb, the first discovered tumor suppressor gene, and maintains Rb in a state of inhibition. Once Rb is mutated or phosphorylated, E2F1 dissociates from the Rb-E2F1 complex and plays a role in the activation of downstream genes^14^. previous studies showed that E2F1 is involved in the PI3K/Akt pathway. On the one hand, suppression of Akt by LY294002 distinctly decreases the level of E2F1 protein expression; on the other hand, E2F1 can transriptionally regulate the expression of the PI3K signaling adaptor protein Gab2 and enhance PI3K activity^15, 16^.

In our study, we demonstrated that *FOXD2-AS1* is up-regulated in bladder cancer tissues and cell lines and that *FOXD2-AS1* is associated with tumor stage and recurrence. *FOXD2-AS1* targeted siRNA affected proliferation and migration/invasion of bladder cancer cells. Further studies revealed that *FOXD2-AS1* was involved in a positive feedback loop with Akt and E2F1. Our study sheds light on the mechanisms of Akt regulation, bladder cancer initiation and bladder cancer development.

## Results

### FOXD2-AS1 is up-regulated in bladder cancer and that associated with tumor stage, tumor recurrence and poor prognosis

**Fig. 1:**
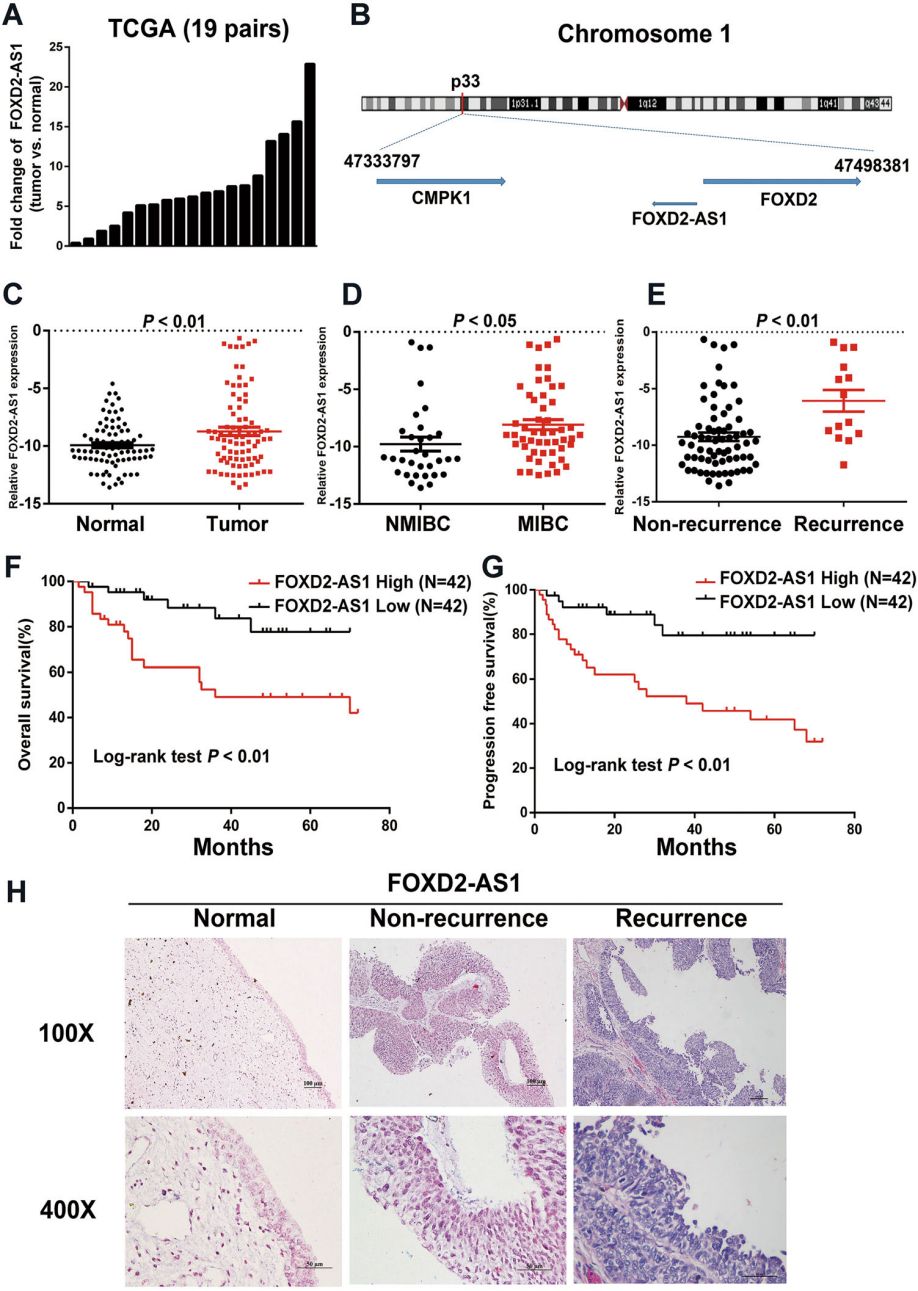
Expression and clinical value of *FOXD2-AS1* in bladder cancer. **a** Expression of *FOXD2-AS1* in 19 pairs of TCGA cancer and adjacent noncancerous samples indicated that *FOXD2-AS1* is obviously up-regulated in bladder cancer. **b** The location of *FOXD2-AS1* on chromosome. **c** qRT-PCR performed in 84 cases showed that *FOXD2-AS1* expression was significantly increased in bladder cacer tissues compared to adjacent noncancerous tissues (*p* < 0.01). **d** *FOXD2-AS1* expression in MIBC patients was obviously higher than in NMIBC patients (*p* < 0.05). **e** Expression of *FOXD2-AS1* was significantly higher in recurrence patients (*p* < 0.01). **f** Kaplan–Meier analysis revealed higher expression of *FOXD2-AS1* is associated with poor prognosis. The *p*-values were assessed by log-rank test (*p* < 0.01). **g** Progression-free time was shorter in *FOXD2-AS1* high expression patients. The *p*-values were assessed by log-rank test (*p* < 0.01). **h** ISH was performed in paraffin-embedded tissues. Expression of *FOXD2-AS1* was extremely higher in relapsed tissues compared to adjacent noncancerous tissues and primary tumor tissues

From The Cancer Genome Atlas (TCGA) database, we found that *FOXD2-AS1* was significantly increased in bladder cancer according to analysis of 19 pairs of cancer and adjacent noncancerous samples (Fig. 1a). *FOXD2-AS1* is located in human chromosome 1p33 and only exists as a 2059 bp transcript (Fig. 1b). To validate the data we found, real-time PCR was performed on 84 cases of bladder cancer and adjacent tissues. As expected, *FOXD2-AS1* was found to exert remarkable clinical significance (Table 1). The statistical analysis demonstrated that *FOXD2-AS1* was significantly increased in bladder cancer tissues and obviously associated with tumor stage and tumor recurrence (Fig. 1c–e). Furthermore, the Kaplan–Meier analysis revealed that *FOXD2-AS1* was associated with overall survival and progression-free survival of bladder cancer patients (Fig. 1f, g).

**Table 1:** Correlation between FOXD2-AS1 expression level and clinical features in bladder cancer

Characteristics	FOXD2-AS1 expression		*P*-value
	High (42)	Low (42)	
*Age*	22	26	0.509
≤65	20	16	
>65			
*Gender*			
Male	34	34	1.000
Female	8	8	
*Grade*			
Low	6	9	0.57
High	36	33	
*T stage*			
Tis/Ta/T1	12	20	0.024*
T2/T3/T4	30	22	
*Lymph node metastasis*			
Yes	14	10	0.469
No	28	32	
*Recurrence*			
Yes	11	3	0.037*
No	31	39	

Chi-square test. *p < 0.05

Finally, we performed an in situ hybridization (ISH) assay to explore the intracellular location and expression discrepancy of *FOXD2-AS1* in bladder cancer tissues versus adjacent noncancerous tissues. The results showed that *FOXD2-AS1* was mainly expressed in the nucleus and that expression was significantly higher in the bladder cancer tissues than in the adjacent normal tissues. Moreover, the recurring patients expressed higher *FOXD2-AS1* than the non-recurring patients (Fig. 1h).

### FOXD2-AS1 promotes bladder cancer cell proliferation and migration and invasion in vitro

**Fig. 2:**
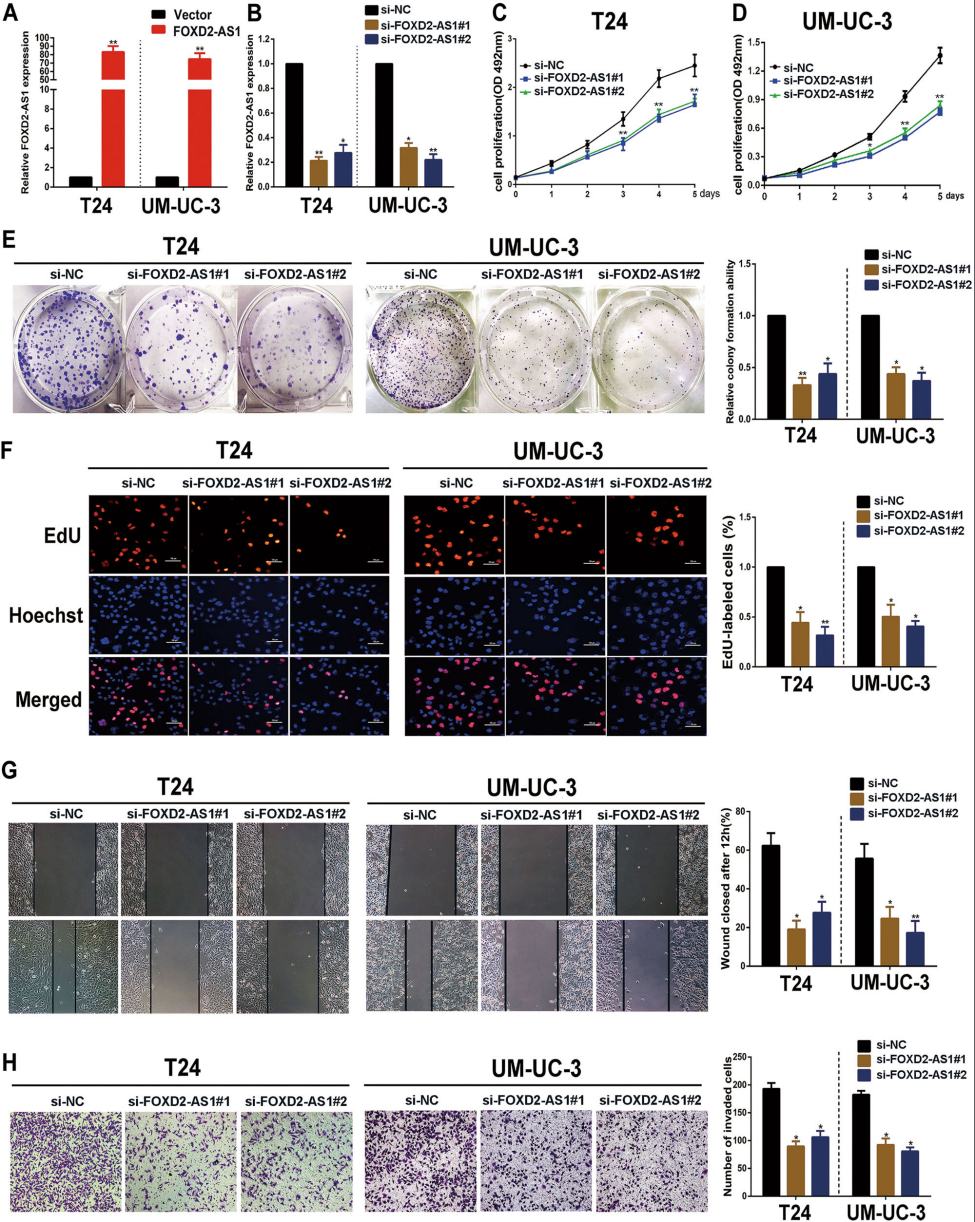
*FOXD2-AS1* promotes proliferation and migration/invasion of bladder cancer cells. **a** Expression alteration of *FOXD2-AS1* was shown in *FOXD2-AS1* overexpression bladder cancer cells. **b** qRT-PCR was used to evaluate expression of *FOXD2-AS1* in two independent siRNAs transfected bladder cancer cells. The figures in A and B showed representative results of three independent experiments. **c** and **d** MTT assays were performed to determine the T24 and UM-UC-3 cell growth capacities after silencing of *FOXD2-AS1* via specific siRNAs transfections. The representative figure of three independent experiments showed that *FOXD2-AS1* obviously promoted bladder cancer cell growth. *p < 0.05, **p < 0.01. **e** *FOXD2-AS1* increased colony formation of bladder cancer cells. Left: representative figure of colony formation assays. Right: Error bars represent the mean ± S.D. from three independent experiments. *p < 0.05, **p < 0.01. **f** The representative figure of EdU assays demonstrated that knockdown of *FOXD2-AS1* decreased the number of cells in the S phase. Error bars represent the mean ± S.D. from three independent experiments. *p < 0.05, **p < 0.01. **g** and **h** Cell migration distances and invasiveness were also (**b**) positively associated with *FOXD2-AS1* expression in T24 and UM-UC-3 cells. Error bars represent the mean ± S.D. from three independent experiments. *p < 0.05, **p < 0.01

Because we validated that *FOXD2-AS1* was associated with bladder cancer progression, we next explored its function in bladder cancer cells. Therefore, two independent siRNAs and expression plasmids of *FOXD2-AS1* were respectively transfected into UM-UC-3 and T24 cells for a function analysis in vitro. The transfection efficiency of *FOXD2-AS1* overexpression plasmids was measured by qRT-PCR (Fig. 2a). similarly, The transfection efficiency of two siRNAs were shown. (Fig. 2b). MTT assay outcomes in both the *FOXD2-AS1*-silenced and *FOXD2-AS1*-overexpressing cell lines revealed that *FOXD2-AS1* was involved in positive regulation of bladder cancer cell viability (Fig. 2c, d, Fig. S1A). Colony formation assays showed the same results as the MTT assays (Fig. 2e, Fig. S1B). Furthermore, the EdU and cell cycle assays demonstrated that *FOXD2-AS1* promoted the G1/S transition (Fig. 2f, Fig. S1C, S1D). These findings indicate that *FOXD2-AS1* has a significant effect on bladder cancer cell proliferation and cell cycle, which are two significant processes in tumor relapse.

In addition, we examined the effect of *FOXD2-AS1* on bladder cancer cell migration and invasion. Wound healing assays revealed that downregulation of *FOXD2-AS1* significantly slowed cell migration, whereas overexpression of *FOXD2-AS1* obviously promoted cell migration (Fig. 2g, Fig. S1E). Matrigel-coated Transwell experiments demonstrated that the invasion capacity of the cells was distinctly weakened with *FOXD2-AS1* depletion (Fig. 2h). The opposite results were observed when *FOXD2-AS1* was up-regulated (Fig. S1F). Altogether, these data powerfully indicate that *FOXD2-AS1* participates in bladder cancer cell proliferation, migration and invasion.

### FOXD2-AS1 promotes the growth of xenograft tumors in vivo

**Fig. 3:**
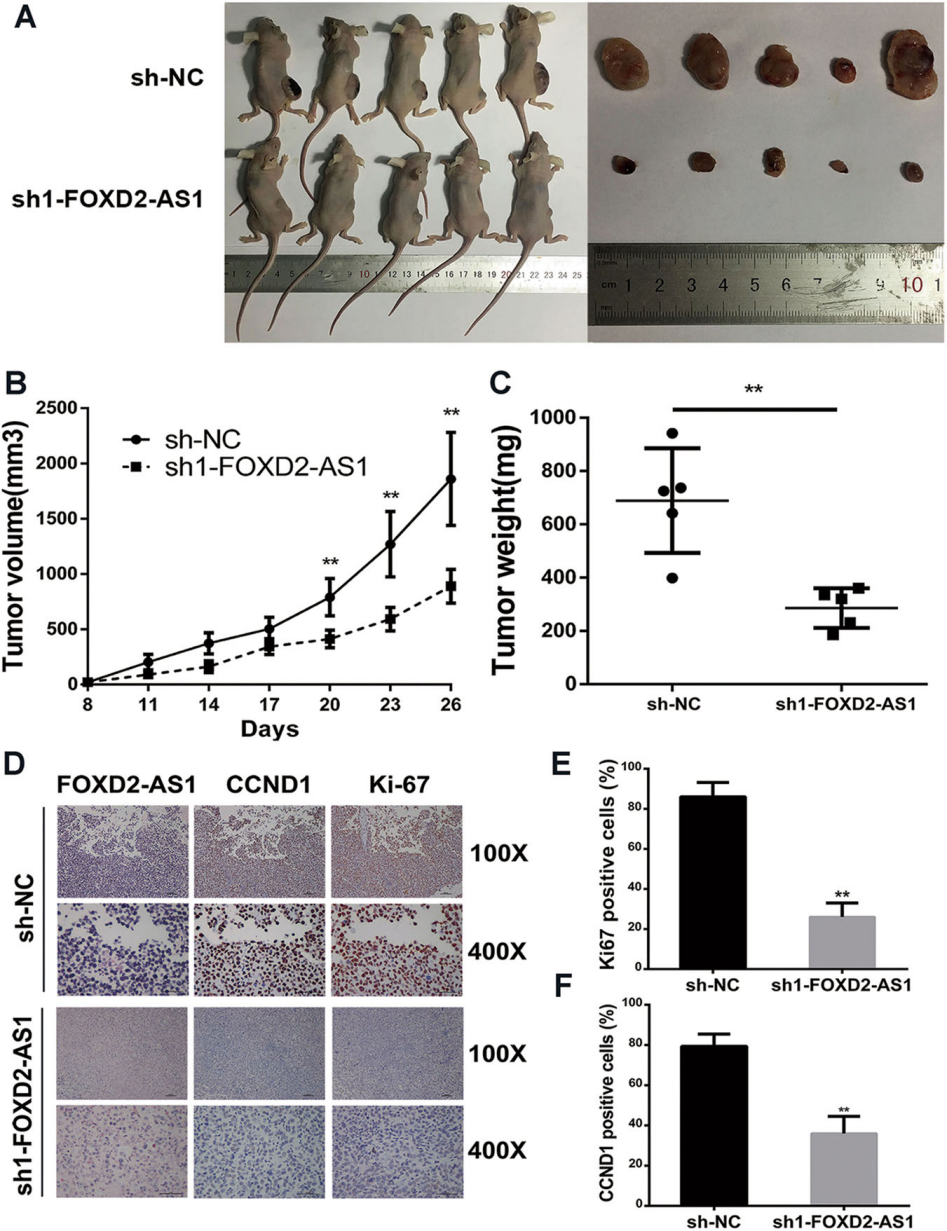
*FOXD2-AS1* exhibits a significant effect on tumorigenesis in vivo. **a** Representative figure of tumor growth in nude mice after injections with sh-NC and sh-*FOXD2-AS1* UM-UC-3 cells. **b** and **c** Tumor volumes and tumor weights were measured for both the sh-NC group and sh-*FOXD2-AS1* group. Two-tailed Student's *t*-tests was used to evaluate the statistical significance of differences of two gruops. **d** Representative figure of ISH for *FOXD2-AS1* and IHC for CCND1 and Ki-67. **e** and **f** statistical results of IHC revealed that *FOXD2-AS1* was associated with proliferation indexes, namely, CCND1 and Ki-67. Error bars represent the mean ± S.D. from three independent experiments. **p < 0.01

Next, we employed a subcutaneous tumor model using female nude mice to evaluate whether *FOXD2-AS1* was required for tumor growth. Briefly, *FOXD2-AS1*-targeted shRNA1or control shRNA was transfected into UM-UC-3 cells. After puromycin screening, the cells were injected into two groups of immunocompromised nude mice. We observed clear and significantly reduced tumor growth in the *FOXD2-AS1*-depleted group (Fig. 3a). The tumor volumes were measured every three days beginning one week after injection. The tumor weights were measured at the end of the experiment on day 30. The results showed that *FOXD2-AS1* silencing markedly reduced the tumor volume and tumor weight (Fig. 3b, c). Furthermore, the ISH assays revealed that *FOXD2-AS1* was extremely low in the *FOXD2-AS1*-knockdown group relative to the control group (Fig. 3d). Immunohistochemistry (IHC) showed that the *FOXD2-AS1* knockdown decreased two proliferation indexes, namely, CCND1 and Ki-67 (Fig. 3e, f).

### Identification of the downstream genes of FOXD2-AS1

**Fig. 4:**
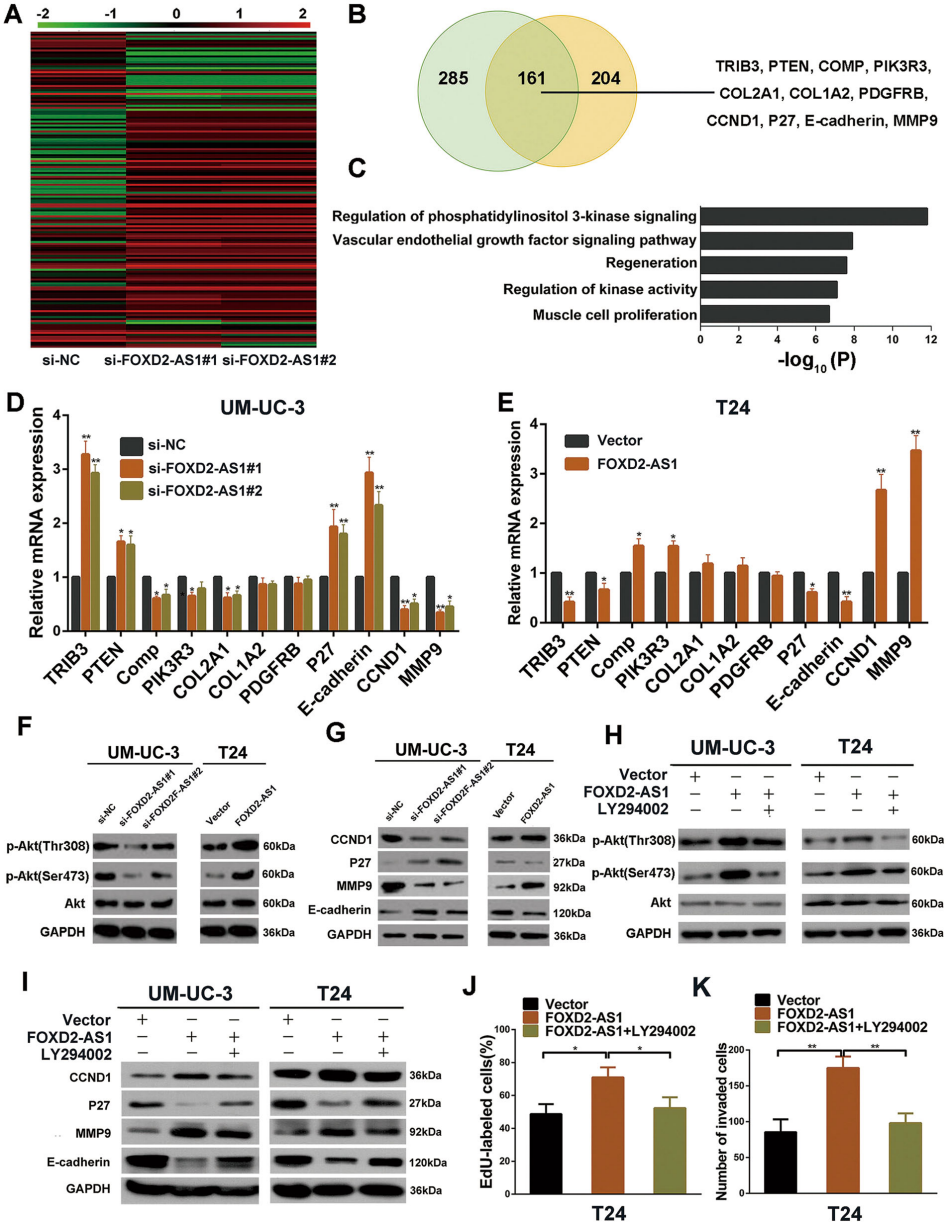
*FOXD2-AS1* promotes bladder cancer cell proliferation, migration and invasion by regulating the Akt signaling pathway. **a** The heatmap showed the differentially expressed genes between the NC and siRNAs groups. **b** Left: Venn diagram showed Co-altered genes of two independent siRNAs targeted to *FOXD2-AS1*. Right: Obviously changed genes involved in phosphatidylinositol 3-kinase signaling were listed. **c** The GO analysis shows that *FOXD2-AS1* is involved in regulation of phosphatidylinositol 3-kinase signaling. **d** and **e** qRT-PCR was employed to verify the results of the microarray analysis in UM-UC-3 and T24 cells. Error bars represent the mean ± S.D. from three independent experiments. *p < 0.05, **p < 0.01. **f** *FOXD2-AS1* overexpression via transfection with pcDNA3.1-*FOXD2-AS1* increases the p-Akt level, whereas siRNA-mediated silencing of *FOXD2-AS1* decreases the p-Akt level. Cell extracts were used for western blotting 72 h after transfection. Immunoblots is the representative image from three independent experiments. **g** Downstream genes of Akt (CCND1, P27, MMP9, and E-cadherin) were detected by western blotting. CCND1 and MMP9 was positively correlated with *FOXD2-AS1*. Conversely, P27 and E-cadherin were negatively correlated with *FOXD2-AS1*. Immunoblots is the representative image from three independent experiments. **h** p-Akt and Akt expression was measured after treated by pcDNA3.1-*FOXD2-AS1* only or pcDNA3.1-*FOXD2-AS1* followed by LY294002. **i** Akt signaling pathway inhibitor LY294002 partly restored the *FOXD2-AS1*-regulated expression levels of CCND1, P27, MMP9 and E-cadherin. Immunoblots is the representative image from three independent experiments. **j** Statistical graph of EdU assays revealed that LY294002 reversed the *FOXD2-AS1*-mediated G1/S transition of bladder cancer cells. Error bars represent the mean ± S.D. from three independent experiments. *p < 0.05. **k** Transwell invasion assay were performed in T24 cells, and LY294002 dramatically weakened the invasiveness mediated by *FOXD2-AS1*. Statistical graph was showed. Error bars represent the mean ± S.D. from three independent experiments. **p < 0.01

To figure out the mechanisms by which *FOXD2-AS1* induces bladder cancer cell proliferation and migration/invasion, we used a RNA microarray to examine the gene expression profiles of UM-UC-3 cells after transfections with either the *FOXD2-AS1*-targeted siRNAs or negative control siRNA. We found 161 co-altered genes for both siRNAs against *FOXD2-AS1* (Fig. 4a, b). Subsequent gene ontology analyses indicated that the genes regulated by *FOXD2-AS1* were enriched for the regulation of phosphatidylinositol 3-kinase signaling and for regulation of kinase activity (Fig. 4c). It is well known that Akt is at the center of these signaling pathways. Therefore, Based on the gene expression profiles, we used qRT-PCR to evaluate changed genes involved in PI3K/Akt signaling pathway in UM-UC-3 and T24 cells (Fig. 4d, e). Next, western blotting was performed to assess the relationship between *FOXD2-AS1* and p-Akt. As expected, the two were positively associated (Fig. 4f). Subsequently, western blotting revealed that downstream genes of Akt were obviously altered in *FOXD2-AS1*-silenced UM-UC-3 cells. The opposite results were detected in *FOXD2-AS1*-OE/T24 cells (Fig. 4g). To confirm that *FOXD2-AS1* affected the cell cycle and EMT through activation of Akt, we evaluated the cell cycle and EMT markers after treating *FOXD2-AS1* overexpression cells with a PI3K inhibitor, LY294002. The alteration of p-Akt and Akt was shown using western blotting (Fig. 4h). As anticipated, the effects of *FOXD2-AS1* were reversed (Fig. 4i). We next tested whether LY294002 could restore the proliferative, migratory and invasive phenotype of *FOXD2-AS1* overexpression cells. Consistently, the EdU assay and Matrigel-coated Transwell assay revealed that Akt inhibition via LY294002 treatment of *FOXD2-AS1* overexpression cells partially impaired *FOXD2-AS1*-induced cell proliferation and invasion (Fig. 4j, k). These results suggest that *FOXD2-AS1*-mediated bladder cancer cell proliferation, migration and invasion depend on phosphorylation of Akt.

### TRIB3 is responsible for FOXD2-AS1-mediated Akt phosphorylation

**Fig. 5:**
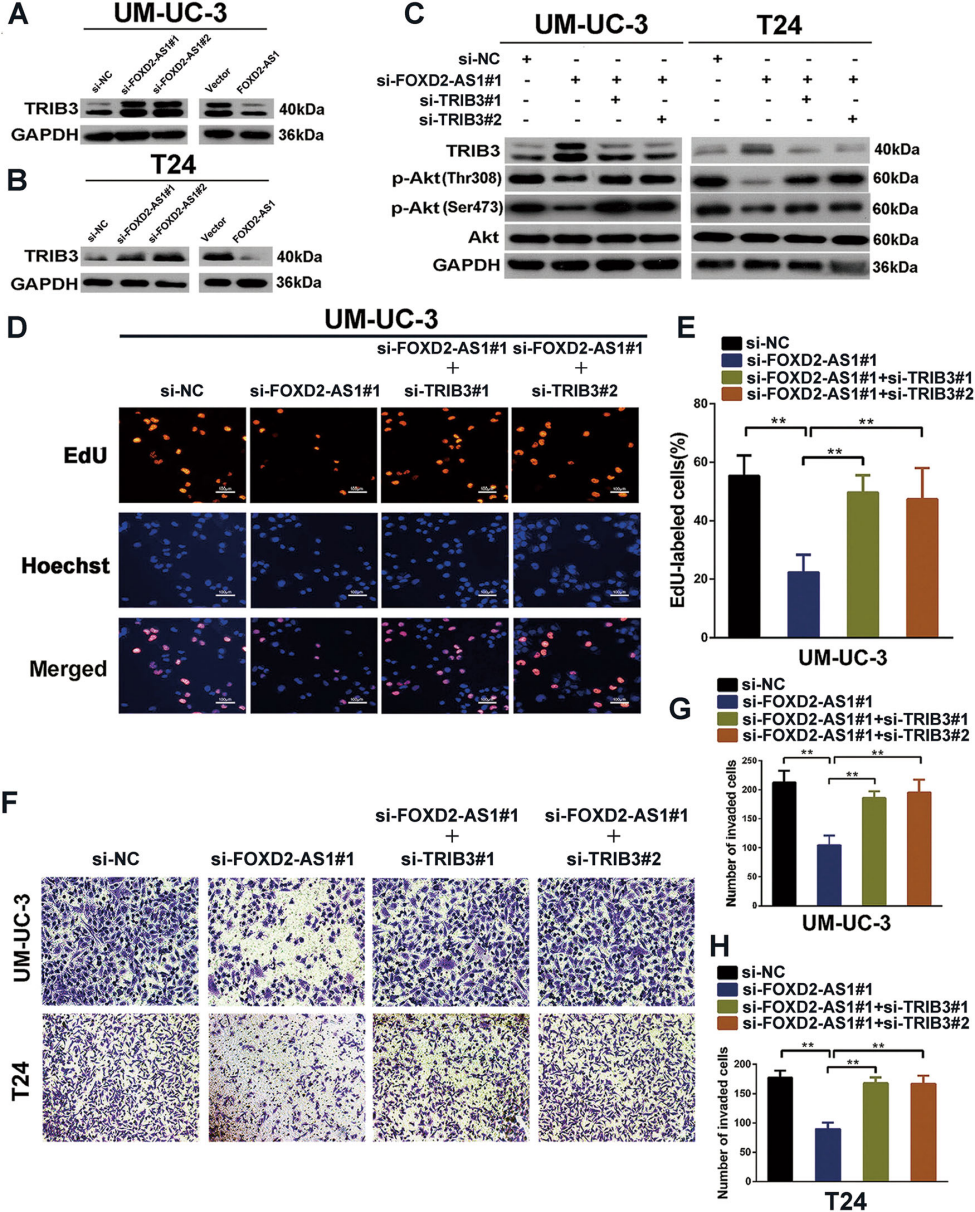
*FOXD2-AS1* regulates Akt phosphorylation and bladder cancer progression via suppressing the expression of TRIB3. **a** and **b** Western blotting revealed that only TRIB3 was significantly changed after ectopic expression or depletion of *FOXD2-AS1*. Immunoblots is the representative image from three independent experiments. **c** As the results of western blotting showed, inhibition of TRIB3 using two independent siRNAs resulted in the rescue of p-Akt in both *FOXD2-AS1*-silenced T24 and UM-UC-3 cells. Immunoblots is the representative image from three independent experiments. **d** and **e** EdU assays revealed that depletion of TRIB3 partially reversed proliferation of *FOXD2-AS1* silencing UM-UC-3 cells. Error bars represent the mean ± S.D. from three independent experiments. **p < 0.01. **f**–**h** The invasiveness properties were restored by silencing TRIB3 in *FOXD2-AS1* depleted bladder cancer cells, as presented by transwell invasion assays. Error bars represent the mean ± S.D. from three independent experiments. **p < 0.01

Next, based on the gene expression profiles and qRT-PCR results above (Fig. 4b–f), we employed western blotting to evaluate the alteration of Akt upstream genes (PIK3R3, COMP and PTEN) after silencing of *FOXD2-AS1*. The results demonstrated that TRIB3, a pseudokinase that is well known to regulate dephosphorylation of Akt, was the most significantly altered gene(Fig. 5a, b, Fig. S1A).However, None of the other genes changed except for PIK3R3. Moreover, western blotting showed that TRIB3 significantly decreased in *FOXD2-AS1* overexpression cells (Fig. 5a, b). To further evaluate the role of TRIB3 in *FOXD2-AS1*-mediated Akt phosphorylation, we co-transfected *FOXD2-AS1* siRNA with TRIB3 siRNAs. The reduction in Akt phosphorylation was clearly rescued by TRIB3 depletion (Fig. 5c). Furthermore, TRIB3 depletion also restored the biological functions of *FOXD2-AS1*-silenced cells (Fig. 5e–i).

### FOXD2-AS1 regulates TRIB3 expression by directly binding to its promoter and recruiting hnRNP L

**Fig 6:**
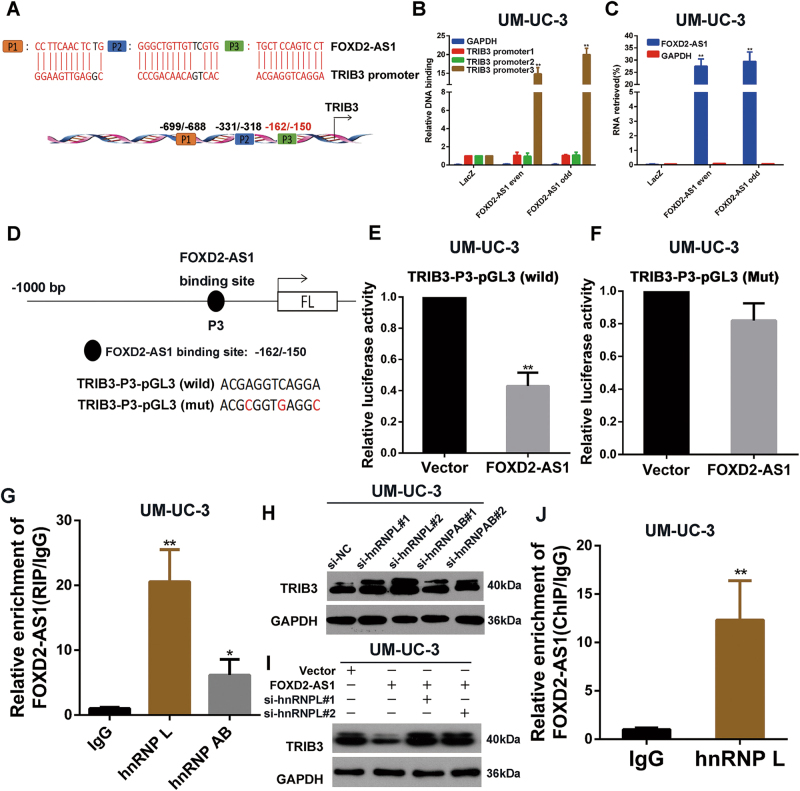
hnRNP L is responsible for *FOXD2-AS1*-mediated regulation of TRIB3. **a** TRIB3 promoter contians three latent binding site with *FOXD2-AS1*. **b** and **c** *FOXD2-AS1* targeted probes and negative Laz probes were used for ChIRP assay. Purified DNA and RNA was analyzed by qPCR and qRT-PCR respectively. The results showed that *FOXD2-AS1* bound to TRIB3-promoter3 and *FOXD2-AS1* probes specifically pulled down *FOXD2-AS1*. Error bars represent the mean ± S.D. from three independent experiments. **p < 0.01. **d** TRIB3 promoter with mutational *FOXD2-AS1* binding site and wild type TRIB3 promoter were cloned into pGL3 reporter vector for dual-luciferase reporter assays. **e** and **f** Dual-luciferase reporter assays showed that pcDNA3.1-*FOXD2-AS1* could impair wild type TRIB3 promoter transcriptional activity compared to control vector. However, no changes were observed in mutation group. Error bars represent the mean ± S.D. from three independent experiments. **p < 0.01. **g** RIP assays revealed that *FOXD2-AS1* bound to hnRNP L and hnRNPAB, IgG was used as negative control. Error bars represent the mean ± S.D. from three independent experiments. *p < 0.05, **p < 0.01. **h** Western blotting showed that depletion of hnRNP L suppressed expression of TRIB3, whereas hnRNPAB silencing showed no changes in TRIB3 expression level. Immunoblots is the representative image from three independent experiments. **i** *FOXD2-AS1* inhibited TRIB3 expression could rescued by silencing of hnRNP L. Immunoblots is the representative image from three independent experiments. **j** ChIP assay showed that hnRNP L significantly bound to TRIB3 promoter. IgG was used as negative control. Error bars represent the mean ± S.D. from three independent experiments. **p < 0.01

Because *FOXD2-AS1* negatively regulates TRIB3 expression, we investigated the molecular mechanisms underlying the correlation between the two. Previous studies have shown that lncRNAs can regulate gene expression at the transcriptional or post-transcriptional level^17, 18^. For transcriptional regulation, lncRNAs can serve to guide transcription factors to a specific sequence by directly forming DNA-RNA complexes with target genes^19–22^. Therefore, we first employed sequence alignment to identify a sequence that was similar to that of the TRIB3 promoter. The results revealed that *FOXD2-AS1* has 3 potential binding sites on the TRIB3 promoter (Fig. 6a). To validate the results, we designed primers according to the predicted binding sites and performed Chromatin Isolation by RNA Purification (ChIRP) assays to explore whether *FOXD2-AS1* possessed an ability to bind to these sites. Among the three promoter fragments, qPCR showed that Biotin-labeled *FOXD2-AS1* could capture TRIB3-promoter3 (−162/−150) (Fig. 6b, c). To clarify whether *FOXD2-AS1* impaired the transcriptional activity of TRIB3 by binding to TRIB3-promoter3, we generated a TRIB3-promoter3 mutation-containing pGL3 reporter vector. The results revealed that the mutant obviously increased luciferase expression compared to the wild-type TRIB3-pGL3 reporter vector after co-transfection with the *FOXD2-AS1* expression plasmid (Fig. 6d–f). Next, CHIRP assays were performed to explore whether other genes involved in PI3K/Akt pathway were regulated by the same molecular mechanism as TRIB3 was. The results showed that *FOXD2-AS1* only bound to PIK3R3-promoter3 and the binding capacity was significantly weaker than TRIB3 promoter (Fig. S1B-F).

Next, we determined which transcription factor was responsible for *FOXD2-AS1*-mediated TRIB3 transcription. Because transcription factors are located in the nucleus, we narrowed down the range of targets to nuclear RNA-binding proteins. Heterogeneous nuclear ribonucleoproteins (hnRNPs) are important RNA-binding proteins in the nucleus. According to previous studies, lncRNAs can both activates and repress transcription of target genes by associating with hnRNPs^23, 24^. Therefore, RNA immunoprecipitation assays were carried out to explore potential hnRNPs that directly interacted with *FOXD2-AS1*. As the results indicated, *FOXD2-AS1* could bind to hnRNP L and hnRNPAB (Fig. 6g). Next, siRNAs targeted to the two hnRNPs were transfected into bladder cancer cells, and expression of TRIB3 was measured by western blotting. Silencing of hnRNP L clearly promoted the expression of TRIB3, whereas hnRNPAB depletion showed no significant effects on TRIB3 expression levels (Fig. 6h). To further verify the role of hnRNP L in *FOXD2-AS1*-mediated TRIB3 regulation, we co-transfected the *FOXD2-AS1* expression plasmid and siRNA targeted to hnRNP L. As expected, hnRNP L depletion partially restored expression of TRIB3 (Fig. 6i). To further explore whether hnRNP L transcriptionally regulate expression of TRIB3, we preformed ChIP assay using hnRNP L antibody. As expected, the results showed hnRNP L directly bound to TRIB3 promoter (Fig. 6j). A ltogether, these data demonstrate that the DNA-RNA complex between *FOXD2-AS1* and the TRIB3 promoter strongly associates with the transcription of TRIB3, which might depend on hnRNP L.

### FOXD2-AS1 forms a positive feedback loop with E2F1

**Fig. 7:**
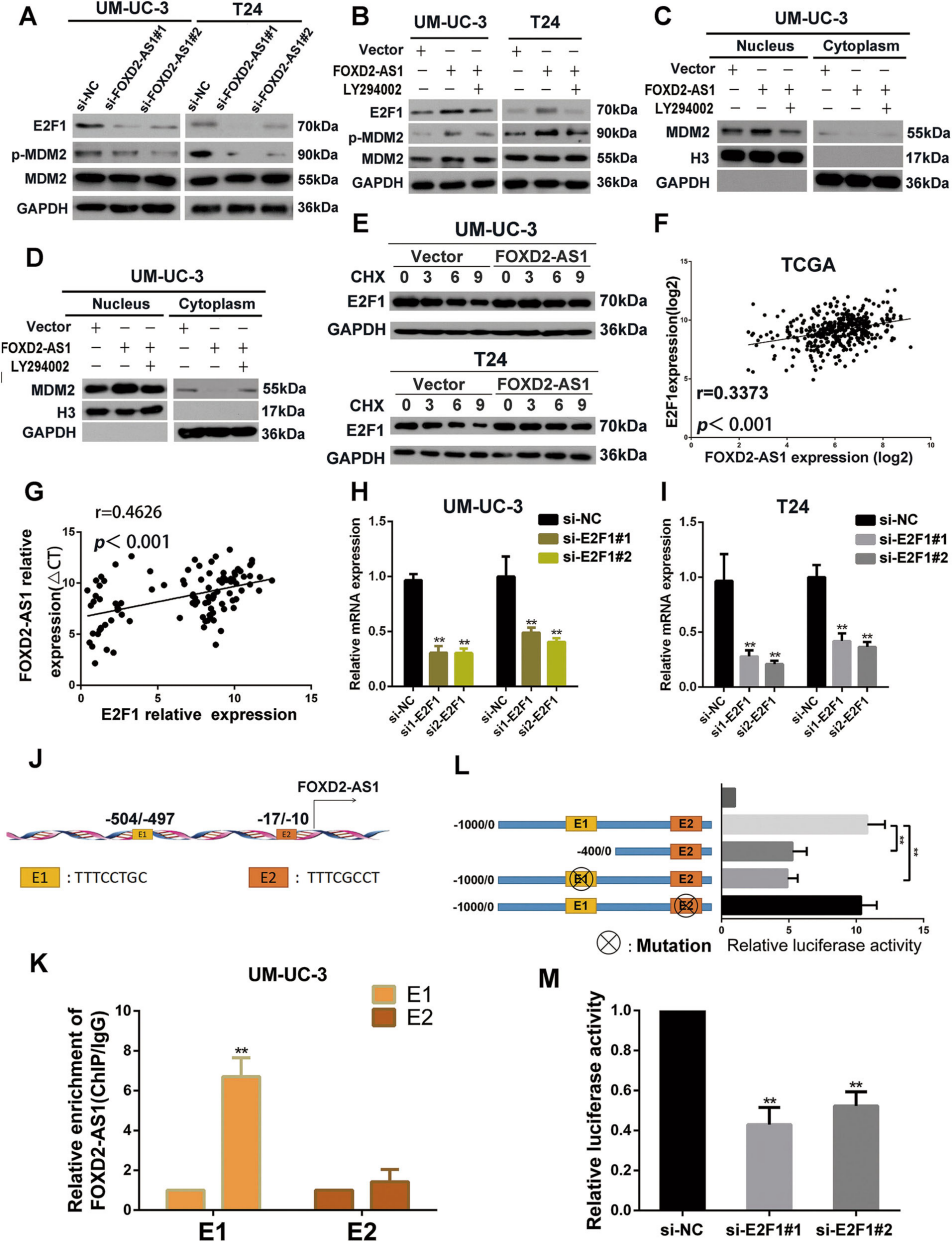
Mutual regulation between *FOXD2-AS1* and E2F1. **a** Western blotting revealed that *FOXD2-AS1* silencing obviously decreased p-MDM2 and E2F1 expression. Immunoblots is the representative image from three independent experiments. **b** LY294002 was used in *FOXD2-AS1* overexpression bladder cancer cells, and the levels of E2F1, p-MDM2 and MDM2 were measured by western blotting after 72 h. *FOXD2-AS1*-mediated p-MDM2 and E2F1 expression could be restored by LY294002. Immunoblots is the representative image from three independent experiments. **c** and **d** Ectopic expression of *FOXD2-AS1* increased MDM2 expression LY294002 significantly restored expression of MDM2 in nucleus in *FOXD2-AS1* overexpression bladder cancer cells. **e** Cells were transfected with *FOXD2-AS1* or corresponding empty vectors, and then exposure to protein synthesis inhibitor CHX (20 μg/ml) for defferent times. E2F1 was detected by western blotting. **f** and **g** *FOXD2-AS1* is positively correlated with E2F1 in bladder cancer tissues which was proved by TCGA data and our own qRT-PCR data. **h** and **i** qRT-PCR showed that E2F1 depletion reduce expression of *FOXD2-AS1* in both T24 and UM-U3 cells. Error bars represent the mean ± S.D. from three independent experiments. **p < 0.01. (J) potential E2F1 binding sites in *FOXD2-AS1* promoter were shown. **k** ChIP assays were carried out in UM-UC-3 cells. The results showed that E2F1 enriched in E1 site of *FOXD2-AS1* promoter. Error bars represent the mean ± S.D. from three independent experiments. **p < 0.01. **l** Dual-luciferase reporter assays revealed that depletion of E1 impaired promoter activity of *FOXD2-AS1*. Mut-E1 impaired E2F1-mediated *FOXD2-AS1* transcription, however, Mut-E2 showed little changes in *FOXD2-AS1* transcriptional activity. Error bars represent the mean ± S.D. from three independent experiments. **p < 0.01. **m** siRNAs targeted to E2F1 significantly reduce the activity of *FOXD2-AS1* promoter reporter. Error bars represent the mean ± S.D. from three independent experiments. **p < 0.01

Our results showed that *FOXD2-AS1* participates in the phosphorylation of Akt and G1/S transition; however, the downstream genes of Akt that link Akt to G1/S transition remain a mystery. MDM2 and E2F1 are well known to be critical downstream regulators of Akt and crucial members which involved in G1/S transition^25–27^. Thus, we used western blotting to assess the expression of p-MDM2, MDM2 and E2F1 in *FOXD2-AS1* silenced cells, we found that p-MDM2 and E2F1 was distinctly altered (Fig. 7a). However, E2F1 mRNA showed no significant change in our downstream microarray. Previous studies demonstrated that inhibition of Akt impaired expression of E2F1 in protein level^15^. In addition, MDM2 directly binds to E2F1 and prolongs the half-life of E2F1 by inhibiting its ubiquitination. This process depends on their co-location in the nucleus, meanwhile, phosphorylation of MDM2 enhances its nuclear localization.^28, 29^. To further explore whether Akt/MDM2 was involved in *FOXD2-AS1*-mediated regulation of E2F1, western blotting was used to evaluate the alteration of p-MDM2 and E2F1 in cells that ectopically expressed *FOXD2-AS1*, which followed by LY294002 treatment. Our results showed that suppression of Akt clearly restored p-MDM2 and E2F1 expression levels compared to the *FOXD2-AS1* ectopically expressing cells (Fig. 7b). Moreover, LY294002 treatment obviously reduced MDM2 expression in nucleus in *FOXD2-AS1* overexpressed UM-UC-3 and T24 cells (Fig. 7c, d). Next, cycloheximide was added to *FOXD2-AS1* overexpressed bladder cancer cells or negative control cells to assess the half-time of E2F1. As the results showed, the half-time of E2F1 was prolonged in *FOXD2-AS1* expression plasmid treated bladder cancer cells than in corresponding empty vectors treated cells (Fig. 7e).

Recent studies showed that a positive feedback loop played an important role in regulation of tumor progression^30, 31^. Therefore, we aimed to clarify whether E2F1 could transcriptionally activated *FOXD2-AS1*. After checking TCGA, we were surprised to find that expression of E2F1 was positively associated with *FOXD2-AS1* (Fig. 7f). The positive correlation between *FOXD2-AS1* and E2F1 was also obeserved in 84 cases of bladder cancer tissues using qRT-PCR (Fig. 7g). Subsequently, qRT-PCR showed that *FOXD2-AS1* expression obviously decreased due to impaired E2F1 expression mediated by siRNAs targeted to E2F1 (Fig. 7h, i). Next, bioinformatics revealed that the *FOXD2-AS1* promoter contained two potential E2F1 binding sites, namely, E1 and E2 (Fig. 7j). Subsequently, ChIP assays were employed to validate whether E2F1 could interact with the promoter of *FOXD2-AS1*. As we predicted, compared to the control IgG, E2F1 was significantly enriched (~6-fold) at the E1 site of *FOXD2-AS1* promoter (Fig. 7k). Next, we used dual-luciferase reporter assays to verify that E2F1 promoted the transcriptional activity of *FOXD2-AS1*. Del-E1, Mut-E1, Mut-E2 and wild type *FOXD2-AS1* promoter were cloned into the pGL3 vector and transfected into UM-UC-3 cells. The results indicated that E1-pGL3 (del) was associated with a decrease in luciferase activity. Further study revealed that E1-pGL3 (Mut) significantly reduced luciferase activity of *FOXD2-AS1* promoter, whereas E2-pGL3 (Mut) showed no significant changes compared to pGL3 vector containing wild type *FOXD2-AS1* promoter (Fig. 7l). Finally, wild type *FOXD2-AS1* promoter was cloned into the pGL3 vector and subsequently co-transfected with E2F1 siRNAs and negative control siRNA into bladder cancer cells. Dual-luciferase reporter assays performed after 48 h showed that siRNA targeted to E2F1 impaired transcriptional activity of *FOXD2-AS1* (Fig. 7m).

## Discussion

Recurrence is the leading causes of poor quality of life of patients with bladder cancer. Recurrence is commonly found in high-risk non-muscle-invasive bladder cancers after resection of the primary tumor^1^. Regrettably, the reasons for recurrence are largely unknown. In our study, we identified that a novel lncRNA *FOXD2-AS1* was associated with bladder cancer recurrence. The underlying mechanism depended on the positive feedback loop among *FOXD2-AS1*, Akt, and E2F1. Our study indicates that *FOXD2-AS1* is likely to become a recurrence predictor in bladder cancer. Besides, we also found a novel regulation pattern of TRIB3, a significant negative regulator of Akt, which was induced by *FOXD2-AS1*.

It is well known that recurrence can be explained partially by the “seeding/implantation” theory, Therefore, proliferation of the surviving tumor cells may play an important role in tumor recurrence^3^. Here, we found that *FOXD2-AS1* expression was associated with bladder cancer recurrence. In order to explored the underlying mechanisms, we performed in vitro and in vivo experiments to evaluate biological function of *FOXD2-AS1*. As expected, *FOXD2-AS1* distinctly promoted proliferation of bladder cancer cells. Previous studies shown that proliferation index Ki-67 could predict recurrence in pT1 urothelial bladder cancer^4, 5^. Our study provided a novel potential recurrence predictor, which might be more specific and we will be concentrate on the ability of *FOXD2-AS1* to predict recurrence of bladder cancer in our further study.

In addition, our study introduced a new regulation pattern of Akt. TRIB3 has been shown to function as an Akt inhibitor by directly interacting with Akt^7^. Our study showed that TRIB3 plays a significant role in bladder cancer suppression, which coincided with previous studies^32, 33^. However, little is known about regulation of TRIB3. In the current study, we proved that *FOXD2-AS1* directly bound to the promoter of TRIB3 and suppressed its transcription for the first time. Moreover, we found that *FOXD2-AS1* could interact with hnRNP L and that hnRNP L knockdown partially restored the silencing of TRIB3 that was caused by up-regulated *FOXD2-AS1*. ChIP assay revealed that hnRNP L directly bound to TRIB3 promoter. All of these results indicated that *FOXD2-AS1* bound to the promoter of TRIB3 and served as a guidance of hnRNP L to regulate expression of TRIB3. It is well established that activation of the PI3K/Akt pathway can be sustained by GFs and is strongly associated with cancer cell growth^6^. For this reason, the PI3K/Akt pathway can be a central factor in tumor relapse. Recently, numerous lncRNAs have been shown to participate in regulation of plenty signaling pathway^34–36^. Our study identified *FOXD2-AS1* was engaged in a novel modulatory mechanism of TRIB3 and PI3K/Akt pathway, which might serve as an essential cause of bladder cancer relapse. hnRNP L belongs to the heterogeneous nuclear ribonucleoprotein (hnRNP) family and serves as an important regulatory protein during different phases of gene expression including transcriptional and post-transcriptional regulation^37–39^. Our study is the first one to reveal that hnRNP L is involved in regulation of TRIB3.

In our further study, we discovered that *FOXD2-AS1* formed a positive feedback loop with Akt and E2F1, which served as a newly identified reason for consistently activated TRIB3/Akt pathway in bladder cancer. Our results determined that *FOXD2-AS1* was involved in regulation of the bladder cancer malignant phenotype. Further analysis showed that *FOXD2-AS1* activated the TRIB3/Akt signaling pathway, which subsequently promoted expression of E2F1, a significant transcription factor in the cell cycle^15^. What we found was consistent with previous studies^15^. In recent years, lncRNAs have been reported to be regulated by multiple transcription factors^10, 11^. However, whether *FOXD2-AS1* is regulated by specific transcription factors remains unknown. E2F1 is a well-known master transcriptional regulator of numerous genes. Several studies have revealed that E2F1 can modulate the expression levels of lncRNAs^20, 40^. To understand the relationship between E2F1 and *FOXD2-AS1* in depth, we identified two binding sites for E2F1 in the *FOXD2-AS1* promoter. Meanwhile, we found that *FOXD2-AS1* and E2F1 were positively correlated according to The Cancer Genome Atlas (TCGA) and our own data. We next employed ChIP assays and dual-luciferase reporter assays to confirm that E2F1 directly bound to the promoter of *FOXD2-AS1* and that E2F1 increased *FOXD2-AS1* expression. Previous studies have shown that thousands of lncRNAs were abnormally expressed in urogenital neoplasms^41^. However, the regulatory mechanisms of the majority of lncRNAs remain obscure. Our study mechanistically explains why *FOXD2-AS1* is up-regulated in bladder cancer and may demonstrate a new modulatory pattern of lncRNAs. Altogether, our results demonstrated that a positive feedback loop comprising *FOXD2-AS1*, Akt and E2F1 was responsible for *FOXD2-AS1*-mediated bladder tumor growth and recurrence. These results may provide a potential preventive and therapeutic method for bladder cancer recurrence by employing a small, targeted fragment to repress *FOXD2-AS1* expression.

In summary, our work shows that *FOXD2-AS1* exhibit strong effects on bladder cancer progression and recurrence thruogh a positive feedback loop with Akt and E2F1. It has the potential to predict recurrence of bladder cancer in the future.

## Materials and methods

### Tissue samples

Bladder cancer specimens were obtained from the Department of Urology, Sun Yat-sen Memorial Hospital. A total of 100 bladder cancer patients who received radical cystectomy were enrolled from August 2011 to July 2016. All patients provided written informed consent. The samples were stored in liquid nitrogen before analyses. The study was approved by the ethics boards of the Sun Yat-sen University Cancer Center, Guangzhou, Guangdong, China.

### Cell culture and transfection

The bladder cancer cell lines were purchased from ATCC (Manassas, VA, USA). T24 and UM-UC-3 cells were cultured in RPMI 1640 (Gibco, Shanghai, China) and DMEM (Gibco, Shanghai, China), respectively. All media contained 10% FBS (Shanghai ExCell Biology, China) and 1% antibiotics (Gibco, 100 U/ml penicillin and 100 μg/ml streptomycin). Cells were cultured in an incubator with 5% CO_2_ at 37℃.

The specific siRNA oligos that were used to target *FOXD2-AS1*, TRIB3 and E2F1 are listed in the supplementary file (Table S1) and were purchased from GenePharma (Suzhou, China). Cells were transfected with 75 nM siRNA using Lipofectamine RNAimax (Invitrogen, USA) according to the manufacturer’s protocol. PcDNA3.1-*FOXD2-AS1* was purchased from IGE Biotechnology (Shanghai, China) and transfected into cells using X-tremeGENE (Roche) following the manufacturer’s instructions.

### RNA isolation and quantitative reverse transcription PCR (qRT-PCR) analysis

Total RNA was extracted from cells and tissue samples using RNAiso Plus reagent (Takara, Dalian, China) based on the manufacturer’s protocol. First-strand cDNA was synthesized from total RNA using PrimeScript RT Master Mix (Takara, Dalian, China), and real-time PCR was performed using SYBR Green PCR kits (Roche) and a LightCycler 96 real-time instrument (Roche). GAPDH served as the internal reference. The 2^−ΔΔCt^ method was used to determine the relative gene expression levels. Relative expression was calculated using the formula of Log 10 ^(2−ΔΔCt)^. The primer sequences are listed in the supplementary file (Table S2).

### Bioinformatics analysis

The RNA-Protein Interaction Prediction (RPISeq) methodology established by the Dobbs and Honavar Laboratories, Iowa State University, was used to predict the RNAbinding protein that could bind to *FOXD2-AS1*. The website is http://pridb.gdcb.iastate.edu/RPISeq/RPIntDB.html. The *FOXD2-AS1* DNA-binding domain was identified with LongTarget, built by Zhu H from Southern Medical University. The website is http://lncrna.smu.edu.cn/show/DNATriplex. The E2F1 binding sites of the *FOXD2-AS1* promoter were predicted using the JASPAR database. The website serves as http://jaspardev.genereg.net/.

### Nuclear-plasma fractionation

For nuclear-plasma fractionation, 1 × 10^7^ cells were digested with trypsin and washed twice with ice cold PBS. The cell pellet was resuspended with 1 ml of RNase-free PBS, 1 ml of buffer C1 (1.28 M sucrose, 40 mM Tris, pH 7.5, 20 mM MgCl_2_, 4% Triton X-100) and 3 ml of RNase-free water. The suspension was incubated on ice for 15 min and then centrifuged for 15 min at 3000 rpm. The supernatant was kept as the plasma portion, and the cell pellet served as the nuclear portion.

### Antibodies and western blotting

The primary antibodies used in this study included anti-Akt, anti-p-Akt, anti-CCND1, anti-E2F1, anti-p-MDM2, and anti-P27 (Cell Signaling Technology, USA) and anti-TRIB3, anti-E-cadherin, anti-MMP9, anti-H3 and anti-GAPDH (Abcam, USA), and anti-MDM2 (Proteintech, USA). The goat anti-rabbit or anti-mouse secondary antibodies were purchased from Kangwei Ltd. Beijing, China.

To determine the protein levels in cells, total protein was extracted using RIPA with a Protease Inhibitor Cocktail. Then, 30 µg of protein was subjected to 10% SDS–PAGE gel electrophoresis and transferred to a polyvinylidene fluoride membrane. After the membrane was blocked with 5% skim milk for 1 h, blots were incubated with primary antibodies. Finally, the blots were incubated with secondary antibodies after being washed with TBST.

### Lentivirus infection

Short hairpin RNA targeting *FOXD2-AS1* was designed according to siRNA1. The pre-packaged lentivirus was purchased from GenePharma (Suzhou, China) and used to infect UM-UC-3 cells following the manufacturer’s instructions.

### Cell proliferation

The methyl thiazolyl tetrazolium (MTT, Promega) colorimetric assay and 5-ethynyl-20-deoxyuridine (EdU) assay kit (RiboBio, Guangzhou, China) were used to assess cell proliferation. For the MTT assay, siRNA-treated or overexpression plasmid-treated bladder cancer cells were incubated in 96-well plates at a density of 1 × 10^3^ cells/well. The absorbance at a wavelength of 490 nm was measured using SpectraMax M5 (Molecular Devices) every day for 5 days.

For the EdU assay, cells were incubated in 24-well plate for 24 h and subsequently transfected with siRNAs or overexpression plasmids. Finally, 100 µl of EdU was added to the cells, and DAPI was used to stain the nuclei. An Olympus laser scanning microscope system was used to obtain the images.

### Colony formation

After transfections with siRNA or the overexpression plasmid for 48 h, cells were seeded into 6-well plates at a density of 1 × 10^3^ cells/well. After 10–14 days, the plates were washed with 1 × PBS, and the cells were fixed with 4% paraformaldehyde for 30 min. Finally, the clones were stained with crystal violet for 30 min, photographed and counted.

### Cell cycle analysis

After transfections with siRNA or the overexpression plasmid for 48 h, cells were digested with trypsin and washed with 1 × PBS. Then, the cells were fixed with 70% ice-cold ethanol. Finally, the cells were treated with 500 µl of RNase A and stained with 50 μg/ml propidium iodide. Measurements were performed and analyzed using the FACSCaliber BD flow cytometer and the BD FACSuite analysis software.

### In situ hybridization (ISH)

In situ hybridization was performed to detect the expression and location of *FOXD2-AS1* in the tissue specimens. On the first day, paraffin slides were dewaxed in xylene and washed with in an ethanol gradient. Afterward, 0.5% Triton-X100 and RNase-free proteinase K were used to expose the nucleic acids before incubation with the probes so that hybridization of the probe with the target gene would be easier. The final step of the first day was an incubation of the specimens with the specific probes for 16 h at 42 ℃. On the second day, a 1% Roche Blocking Solution was used to incubate the specimens for 30 min to block the nonspecific binding sites. Then, the specimens were incubated with horseradish peroxidase (HRP)-conjugated anti-DIG antibodies at 4 ℃ overnight to detect the specific gene. On the third day, the BCIP/NBT color-substrate solution was used to stain the specimens. The nuclear was dyed red by Nuclear Fast Red solution. The blue staining represent positive signal of objective probe. Positive control (U6) and negative control (Scramble) was shown in the supplementary file (Fig. S3). Finally, images were captured and analyzed.

### Immunohistochemistry (IHC)

Paraffin slides were dewaxed in xylene and washed with in an ethanol gradient. Afterward, tissues were incubated in 1% hydrogen peroxide for10 min to remove endogenous peroxidase and boiled in tris-EDTA (pH = 9.0) for 15 min. Next, tissues were incubated with primary antibodies overnight at 4 ℃. Finally, HRP conjugated mouse or rabbit secondary antibodies were added to the tissues and incubated for 30 min.

### Tumorigenicity assays in nude mice

All experimental procedures involving animals were performed per the Guide for the Care and Use of Laboratory Animals (NIH publication No. 80-23, revised 1996) and complied with the institutional ethical guidelines for animal experiments. *FOXD2-AS1* stably knocked down and NC-transfected cells (5 × 10^6^) were injected into the left and right outer flanks of NOD/SCID female mice, respectively. Each group contained 5 mice, and measurements of the tumor volumes and weights were performed every 3 days. *FOXD2-AS1* expression in the tumor tissue was analyzed by in situ hybridization.

### RNA immunoprecipitation (RIP)

RNA immunoprecipitation (RIP) assays were performed using a Magna RIP™ RNA-Binding Protein Immunoprecipitation Kit (Millipore, Massachusetts, USA) following the manufacturer’s instructions. A total of 1 × 10^7^ T24 or UM-UC-3 cells were lysed with RIP lysis buffer and stored at -80℃. Cell extracts were incubated with target antibodies or negative-control normal mouse IgG. Finally, proteins were digested using Proteinase K, and RNA was isolated and used for qRT-PCR.

### Chromatin isolation by RNA purification (ChIRP)

ChIRP assays were performed using a Magna ChIRP^TM^ Chromatin Isolation by RNA Purification Kit (Millipore, Massachusetts, USA) following the manufacturer’s instructions. First, 1 × 10^7^ cells (100 mg) were collected for each reaction (NC, odd and even). A total of 100 mg of cells were lysed using 1 ml of Complete Lysis Buffer, and the DNA fragments were then sheared into small pieces by ultrasonication. Second, the supernatant was incubated with probes and Complete Hybridization Buffer at 37 ℃ for 4 h. Third, the probes combined with DNA were mixed with streptavidin magnetic beads (Millipore, Massachusetts, USA) and washed with wash buffer. Finally, RNA and DNA were isolated and purified for qRT-PCR. Probes are listed in the supplementary file (Table S3).

### Chromatin immunoprecipitation assay (ChIP)

ChIP assays were performed using the Magna ChIP assay kit (Millipore, Massachusetts, USA) according to the manual. Briefly, 1 × 10^7^ cells were fixed with 1% formaldehyde for 10 min at room temperature. Then, 10 × glycine was added to the solution to neutralize the excess formaldehyde. Next, sonication was used to shear the DNA into 200-500-nt fragments. Antibodies targeting E2F1, hnRNPL and IgG were used for each assay. qRT-PCR was used to evaluate the enrichment of E2F1 and IgG. The primers involved in these assays are listed in the supplementary file (Table S2).

### Dual-luciferase reporter assay

The *FOXD2-AS1* promoter contains three E2F1 binding sites, namely, E1 (-901/-894), E2 (-504/-497) and E3 (−17/−10), respectively. Different deleption *FOXD2-AS1* promoter fragments were inserted upstream of the promoter of the luciferase gene in the pGL3 vector. The TRIB3 promoter was directly inserted into the pGL3 vector. UM-UC-3 cells were seeded into a 6-well plate and co-transfected with the above constructs along with the *FOXD2-AS1* expression plasmid or E2F1 expression plasmid, and the pGL3 vector was used as a negative control. A reporter plasmid containing Renilla luciferase was used as the standard reference. After 48 h of incubation, the luciferase activities were measured and analyzed with the Dual-Luciferase Reporter Assay System (Promega, USA).

### Statistical analysis

All statistical data were analyzed using the SPSS 20.0 software (IBM, SPSS, Chicago, IL, USA) and GraphPad Prism 6. Two-tailed Student's *t*-tests or Wilcoxon tests were used for comparisons between 2 groups according to the different data types. One-way ANOVA with Bonferroni’s test was used for multiple comparisons. The Kaplan–Meier method with the log-rank test was applied for OS and RFS. Cox regression analysis was performed to evaluate the survival data. Statistical significance is denoted by a *p* value < 0.05.

## Electronic supplementary material

Supplementary Figures

Supplementary tables
