# Supplementary material for: The long non-coding RNA FOXD2-AS1 promotes bladder cancer progression and recurrence through a positive feedback loop with Akt and E2F1
Source: Cell Death Dis. 2018 Feb 14;9(2):233. doi: 10.1038/s41419-018-0275-9 (PMC5833400; doi:10.1038/s41419-018-0275-9)
Supplement: Supplementary file 1 — Supplementary Figures [file 41419_2018_275_MOESM1_ESM.docx]

**
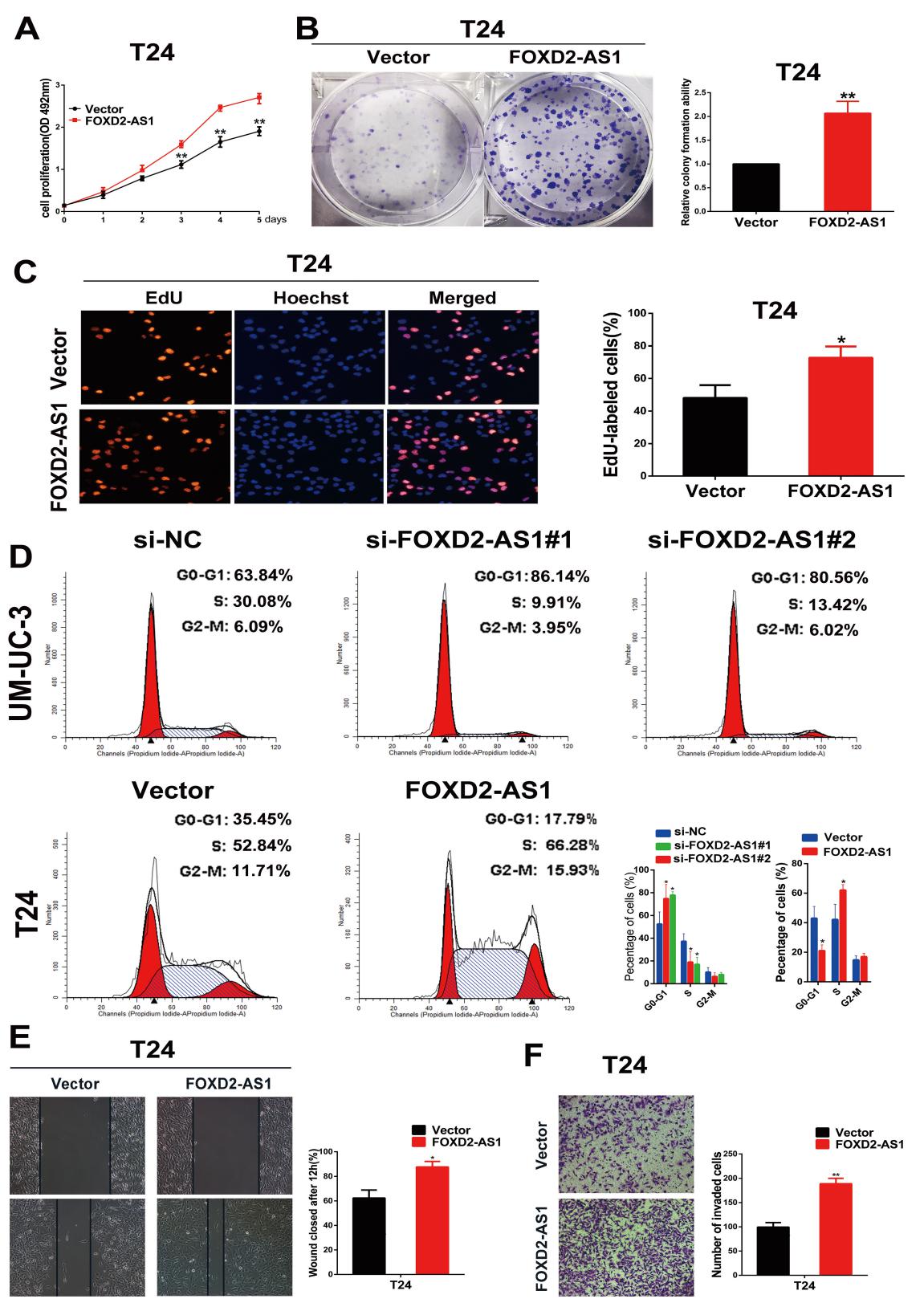
**

**Supplementary Figure S1. *FOXD2-AS1* promotes bladder cancer cell proliferation, migration and invasion.** A. MTT assay revealed that up-regulation of *FOXD2-AS1* promotes proliferation of T24 cells. The experiment was repeated 3 times. B. *FOXD2-AS1* increased colony formation of bladder cancer cells. Error bars represent the mean±S.D. from three independent experiments. **p<0.01. C. The representative figure of three independent EdU assays demonstrated that *FOXD2-AS1* promoted T24 cells G1/S transition. Error bars represent the mean±S.D. from three independent experiments. *p<0.05. D. Cell cycle analysis revealed that *FOXD2-AS1* promoted G1/S transition. Error bars represent the mean±S.D. from three independent experiments. *p<0.05. E and F. Wound healing assays and transwell invasion assays showed that cell migration distances and invasiveness were positively associated with *FOXD2-AS1* expression in T24 cells. Error bars represent the mean±S.D. from three independent experiments. *p<0.05, **p<0.01.


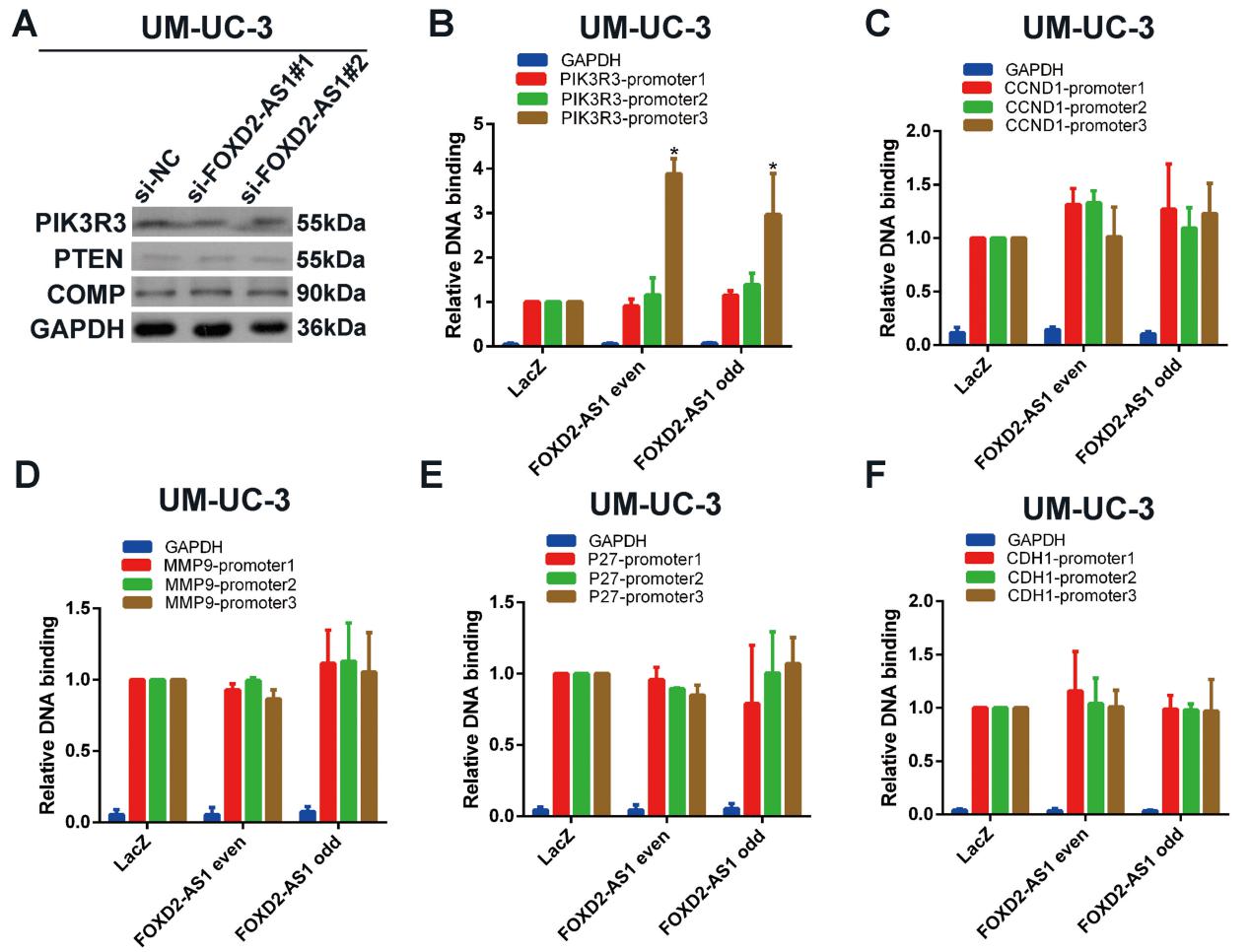


**Supplementary Figure S2. *FOXD2-AS1* regulates PI3KR3 by directly binding to its promoter.** A. western blotting showed that silencing of *FOXD2-AS1* reduced expression of PIK3R3. The experiment was repeated 3 times. (B-F) CHIRP assays was used to assess whether *FOXD2-AS1* directly bound to PIK3R3, CCND1, MMP9, P27 and CDH1 promoters. The results revealed that *FOXD2-AS1* only bound to PIK3R3 promoter. Error bars represent the mean±S.D. from three independent experiments. *p<0.05.

**Supplementary Figure S3.**
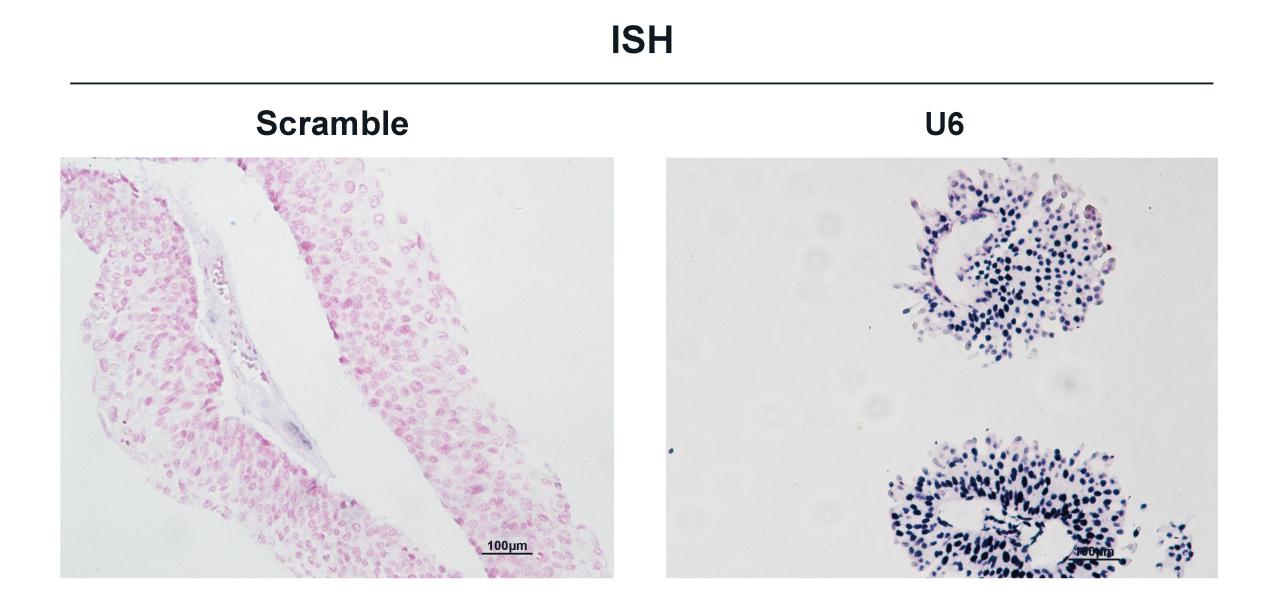
Nagetive control (left) and positive control (right) of ISH assays.
