# Supplementary material for: The long non-coding RNA FOXD2-AS1 promotes bladder cancer progression and recurrence through a positive feedback loop with Akt and E2F1
Source: Cell Death Dis. 2018 Feb 14;9(2):233. doi: 10.1038/s41419-018-0275-9 (PMC5833400; doi:10.1038/s41419-018-0275-9)
Supplement: Supplementary file 2 — Supplementary tables [file 41419_2018_275_MOESM2_ESM.docx]

| **Genes** | **Sense（5’-3’）** | **Antisense (5’-3’)** |
| --- | --- | --- |
| Si1-*FOXD2-AS1*  Si2-*FOXD2-AS1*  Si1-E2F1  Si2-E2F1  Si1-hnRNPL  Si2-hnRNP L  Si1-hnRNPAB  Si2-hnRNPAB | GCGAAGAGUACGUUGCUAUTT  GUUCGAGAGUGAAUUUACATT  GCUGGACCACCUGAUGAAUTT  GGACUCUUCGGAGAACUUUTT  CACUGGUGGAGUUUGAAGATT  CCCAUUUAUUCGAUCACCATT  CAAGAGGGUUUGGGUUUAUTT  GGUAGUACAAACUACGGCATT | AUAGCAACGUACUCUUCGCTT  UGUAAAUUCACUCUCGAACTT  AUUCAUCAGGUGGUCCAGCTT  AAAGUUCUCCGAAGAGUCCTT  UCUCCAAACUCCACCAGUGTT  UGGUGAUCGAAUAAAUGGGTT  AUAAACCCAAACCCUCUUGTT  UGCCGUAGUUUGUACUACCTT |

**Supplementary data**

**Supplementary Table S1. siRNA oligos used in this study.**

**Supplementary Table S2. Primers used in this study.**

| **Genes** | **Forward Primer (5'→3')** | **Reverse Primer (5'→3')** |
| --- | --- | --- |
| *FOXD2-AS1*  GAPDH  TRIB3  PTEN  Comp  PIK3R3  MMP9  COL2A1  COL1A2  PDGFRB  P27  E-cadherin  CCND1  *FOXD2-AS1* promoter1  *FOXD2-AS1* promoter2  TRIB3 promoter1  TRIB3 promoter2  TRIB3 promoter3 | CCGCGTAAGCCTCATAGAAG  GAGTCAACGGATTTGGTCGT  CCAAACCTTCAGTGCCTTCC  CATAACGATGGCTGTGGTTG  TGTGGGTTACACTGCCTTCA  AGTCTGTTTCATTGGGGTTT  CTTCTACGGCCACTACTGTG  TCTACCCCAATCCAGCAAAC  TTGACCCTAACCAAGGATGC  GAGGAATCCCTCACCCTCTC  CAGGTAGTTTGGGGCAAAAA  TGGACAGGGAGGATTTTGAG  CGTGGCCTCTAAGATGAAGG  TGAGACTAGCCAGGGCACTT  CTCTCCTCCCTTCCAAGGTT  TCCTTTGAGCCCAGAGTCAG  GGAAACCGAGGCTCAGAAGA  GCCTGGGCGACAGAGCAAGA | GGGAGTAGGGTGAGGAAAGG  GACAAGCTTCCCGTTCTCAG  CACCTGATAAGCACCCAAGC  CCCCCACTTTAGTGCACAGT  AAGCTGGAGCTGTCCTGGTA  TTCAGAGGCTTCCAAATACA  CCCATCCTTGAACAAATACA  GTTGGGAGCCAGATTGTCAT  CAGTTCTTGGCTGGGATGTT  GACAAATGTGCAACCACCTG  TGTGTTTACACAGCCCGAAG  GGCGTTGTCATTCACATCAG  CCACTTGAGCTTGTTCACCA  ACTCCCCTTCCCCAGTGTAG  CCGGTAGGCGAAAGAAGTAA  GAAGTGGACATGCGGGAATC  GTCTCGATCTCCTGACCTCG  GCAGACATCAGCCACGGACT |

**Supplementary Table S3. Probes used in ChIRP assay.**

| **Probes** | **Sequences** |
| --- | --- |
| 1 | CAGAGACGCTGTAACCAAGA |
| 2 | GCCGAGAACAGCTCATTTAT |
| 3 | TAGACAGCTATCTCGCTTTG |
| 4 | ATTCACTCTCGAACTTTGCC |
| 5 | GGAACTCTGTGATCTTCAGG |
| 6 | AGAAACCCACAAGAGCGCAC |
| 7 | CTTCAGACTCTGGGGGGAAG |
| 8 | CTCAGAAGGGTTACACGTGA |
| 9 | CTGACTCTGTGTGGATGAGA |
| 10 | CTTTTTGGAGAAGAGCAGGC |
